# Diverse biological effects of glycosyltransferase genes from Tartary buckwheat

**DOI:** 10.1186/s12870-019-1955-z

**Published:** 2019-08-05

**Authors:** Panfeng Yao, Renyu Deng, Yunji Huang, Simon Stael, Jiaqi Shi, Guanlan Shi, Bingbing Lv, Qi Li, Qixin Dong, Qi Wu, Chenglei Li, Hui Chen, Haixia Zhao

**Affiliations:** 1 College of Life Science, Sichuan Agricultural University, Ya’an, Sichuan Province, People’s Republic of China; 2 Department of Plant Biotechnology and Bioinformatics, Ghent University, Ghent, Belgium; 3 VIB-UGent Center for Plant Systems Biology, Ghent, Belgium; 4 Department of Biomolecular Medicine, Ghent University, Ghent, Belgium; 5 VIB-UGent Center for Medical Biotechnology, Ghent, Belgium

**Keywords:** Flavonoids glycosyltransferase, Anthocyanins, Development, Tartary buckwheat

## Abstract

**Background:**

Tartary buckwheat (*Fagopyrum tataricum*) is an edible cereal crop whose sprouts have been marketed and commercialized for their higher levels of anti-oxidants, including rutin and anthocyanin. UDP-glucose flavonoid glycosyltransferases (UFGTs) play an important role in the biosynthesis of flavonoids in plants. So far, few studies are available on UFGT genes that may play a role in tartary buckwheat flavonoids biosynthesis. Here, we report on the identification and functional characterization of seven UFGTs from tartary buckwheat that are potentially involved in flavonoid biosynthesis (and have varying effects on plant growth and development when overexpressed in *Arabidopsis thaliana*.)

**Results:**

Phylogenetic analysis indicated that the potential function of the seven FtUFGT proteins, FtUFGT6, FtUFGT7, FtUFGT8, FtUFGT9, FtUFGT15, FtUFGT40, and FtUFGT41, could be divided into three *Arabidopsis thaliana* functional subgroups that are involved in flavonoid biosynthesis of and anthocyanin accumulation. A significant positive correlation between *FtUFGT8* and *FtUFGT15* expression and anthocyanin accumulation capacity was observed in the tartary buckwheat seedlings after cold stress. Overexpression in *Arabidopsis thaliana* showed that *FtUFGT8*, *FtUFGT15*, and *FtUFGT41* significantly increased the anthocyanin content in transgenic plants. Unexpectedly, overexpression of *FtUFGT6,* while not leading to enhanced anthocyanin accumulation*,* significantly enhanced the growth yield of transgenic plants. When wild-type plants have only cotyledons, most of the transgenic plants of *FtUFGT6* had grown true leaves. Moreover, the growth speed of the *oxFtUFGT6* transgenic plant root was also significantly faster than that of the wild type. At later growth, *FtUFGT6* transgenic plants showed larger leaves, earlier twitching times and more tillers than wild type, whereas *FtUFGT15* showed opposite results.

**Conclusions:**

Seven *FtUFGTs* were isolated from tartary buckwheat. *FtUFGT8*, *FtUFGT15*, and *FtUFGT41* can significantly increase the accumulation of total anthocyanins in transgenic plants. Furthermore, overexpression of *FtUFGT6* increased the overall yield of *Arabidopsis* transgenic plants at all growth stages. However, *FtUFGT15* shows the opposite trend at later growth stage and delays the growth speed of plants. These results suggested that the biological function of *FtUFGT* genes in tartary buckwheat is diverse.

**Electronic supplementary material:**

The online version of this article (10.1186/s12870-019-1955-z) contains supplementary material, which is available to authorized users.

## Background

Flavonoids, including flavonols, anthocyanins, isoflavones, and proanthocyanidins, are secondary metabolites found in plants. Among them, anthocyanins are important as flower pigments, ultraviolet-B (UV-B) protectants, and signaling molecules between plants and human beings that include regulators of auxin transport, age retardation, and coronary disease inhibition [1]. Flavonols, colorless co-pigments, affect the brightness and brilliance of colors and play vital roles in pollen germination [2]. Because of these properties, the potential applications of flavonoids have drawn much research and commercial attention in recent years [3].

The biosynthesis of flavonoids involves a branch of the phenylpropanoid metabolic pathway and has been well studied in various plants, including *Petunia hybrida*, *Arabidopsis thaliana,* and *Zea mays* [4, 5]. Additionally, glycosylation is the final step and serves various functions in plant flavonoid metabolism. For instance, glycosylation can increase the stability and solubility of the acceptor molecule and affects their subcellular localization and biological functions [6, 7]. In a wider perspective, glycosylation is also involved in cellular homeostasis and plant growth or may regulate the detoxification of exogenous toxins [8, 9]. The enzymes that catalyze the formation of glycoside are known as uridine diphosphate (UDP): flavonoid glycosyltransferases (UFGTs), which transfer UDP-activated sugar moieties to the low-molecular-weight acceptor aglycone [10, 11]. Additionally, plant UGTs play important roles in regulating the activity of plant hormones. For example, *Arabidopsis* UGT73C5 glycosylates steroid hormone brassinosteroids and reduces their bioactivity [12]. Gain or loss of these UGTs in *Arabidopsis* can perturb hormone levels and substantially affect seed production, root growth, leaf size and shape, shoot height, shoot branching, and flowering time [13]. Phylogenetic analyses of the UFGTs showed that these enzymes can be classified into three different groups-UF3GT, UF5GT, and UF7GT-based on the regioselectivity of flavonoid glycosylation [14, 15]. Additionally, such incongruence between the phylogenetic position and substrate specificities has been found in other UGTs, including grape *VLOGT2*, onion *UGT73G1* and *UGT73J1* [16]. These studies support the proposition that the functions and specificities of UGTs are perhaps not accurately determined based on their protein sequences alone [17]. Therefore, the biological functions of UFGTs in plants are complex and diverse, and the coupling of phylogenetic analyses with experimental analyses is normally regarded as the most efficient and accurate method to identify UGT proteins.

Tartary buckwheat (*Fagopyrum tataricum*) is an edible cereal crop whose sprouts have been marketized and commercialized for their higher levels of anti-oxidants, including rutin and anthocyanin. However, little research has been conducted on *FtUFGT* genes from tartary buckwheat involved in the anthocyanin synthesis pathway. Thus far, only one article has reported on the UFGT family of tartary buckwheat, and the results indicated that *FtUFGT1*, *FtUFGT2*, and *FtUFGT3* can convert cyanidin to cyanidin 3-Oglucoside [18]. Therefore, further cloning and characterization of UFGTs proteins are important works to reveal their functions in tartary buckwheat. In this study, seven new *FtUFGT* genes were isolated from tartary buckwheat, and their promoters and response to light and cold stress, as triggers of increased anthocyanin accumulation, were analyzed. Additionally, heterologous expression in *Arabidopsis thaliana* was employed to investigate their function in plant.

## Results

### Screening of *FtUFGT* genes in tartary buckwheat

To further study the *UFGTs* involved in flavonoid synthesis in tartary buckwheat, we used UDP-glucose: flavonoid 3-O-glucosyltransferase as a probe to screen the transcriptome of tartary buckwheat [19]. We obtained 41 *UFGT* Unigenes by scanning the transcriptome database. For further analysis of the function of these genes, we selected 34 *Arabidopsis thaliana* UGT genes that were previously used to construct a phylogenetic tree [20] (Additional file 1: Figure S1). As observed in a phylogenetic tree, UFGT in buckwheat was divided into 12 subfamilies (A, B, C, D, E, F, G, H, J, L, M, and P) and had different biological functions. On this basis, we selected seven unrevealed UFGT genes that were related to flavonoid synthesis and had a relatively high level of expression in the transcriptome for further study. All seven full-length UFGT genes were named, *FtUFGT6*, *FtUFGT7*, *FtUFGT8*, *FtUFGT9*, *FtUFGT15*, *FtUFGT40*, and *FtUFGT41*, and were submitted to GenBank with accession numbers MG267387-MG267393. The genomic structure of these seven genes was analyzed by comparing their gDNA and cDNA sequences. There are three forms of intron-exon structures in these seven *FtUFGT* genes: type I contained three exons and two introns (*FtUFGT41*), type II contained two exons and one intron (*FtUFGT15*), and type III contained only one exon (*FtUFGT6*, *FtUFGT7*, *FtUFGT8*, *FtUFGT9*, and *FtUFGT40*) (Additional file 2: Figure S2).

### Sequence analyses of *FtUFGTs*

**Fig. 1:**
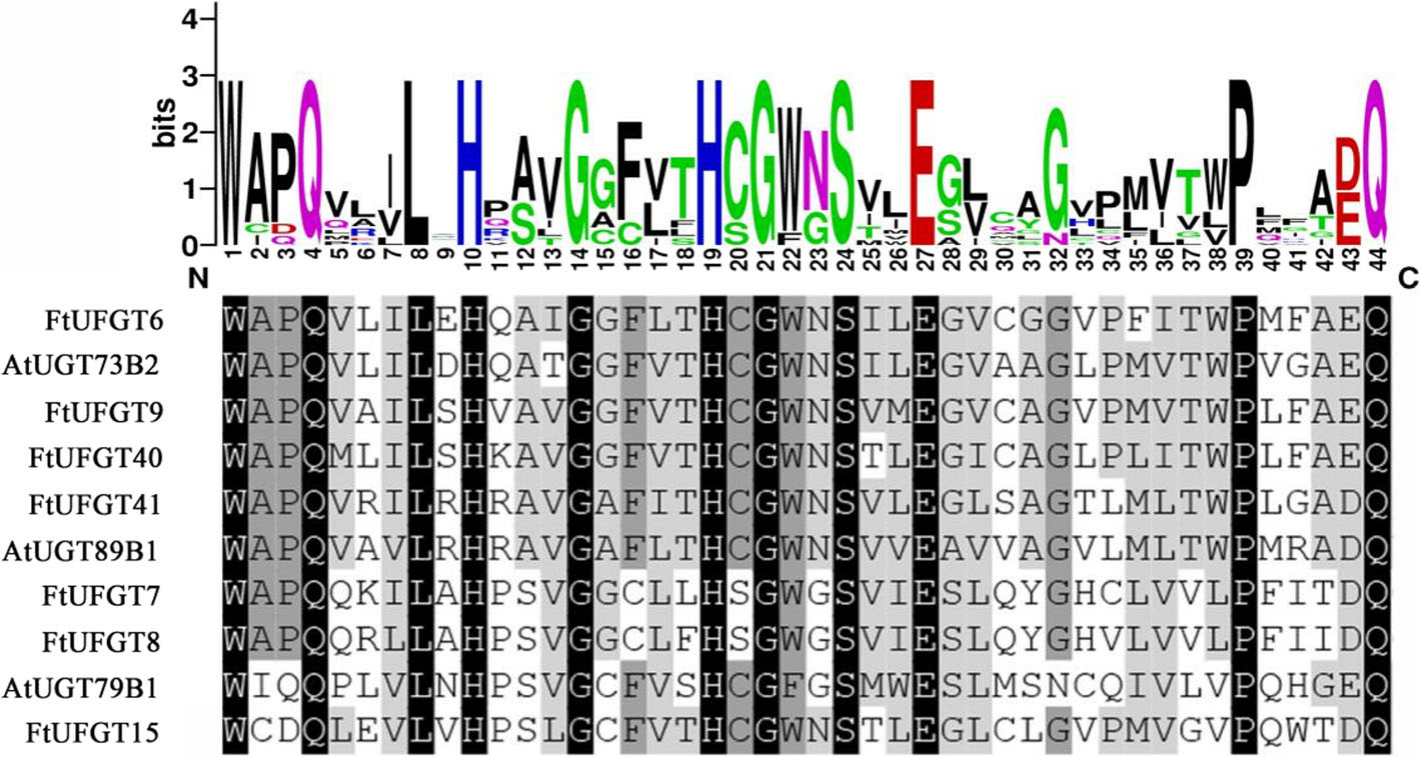
Sequence conservation analysis of the PSPG motif in the seven tartary buckwheat FtUFGT proteins. The multiple sequence alignment was performed using a ClustalX program

**Fig. 2:**
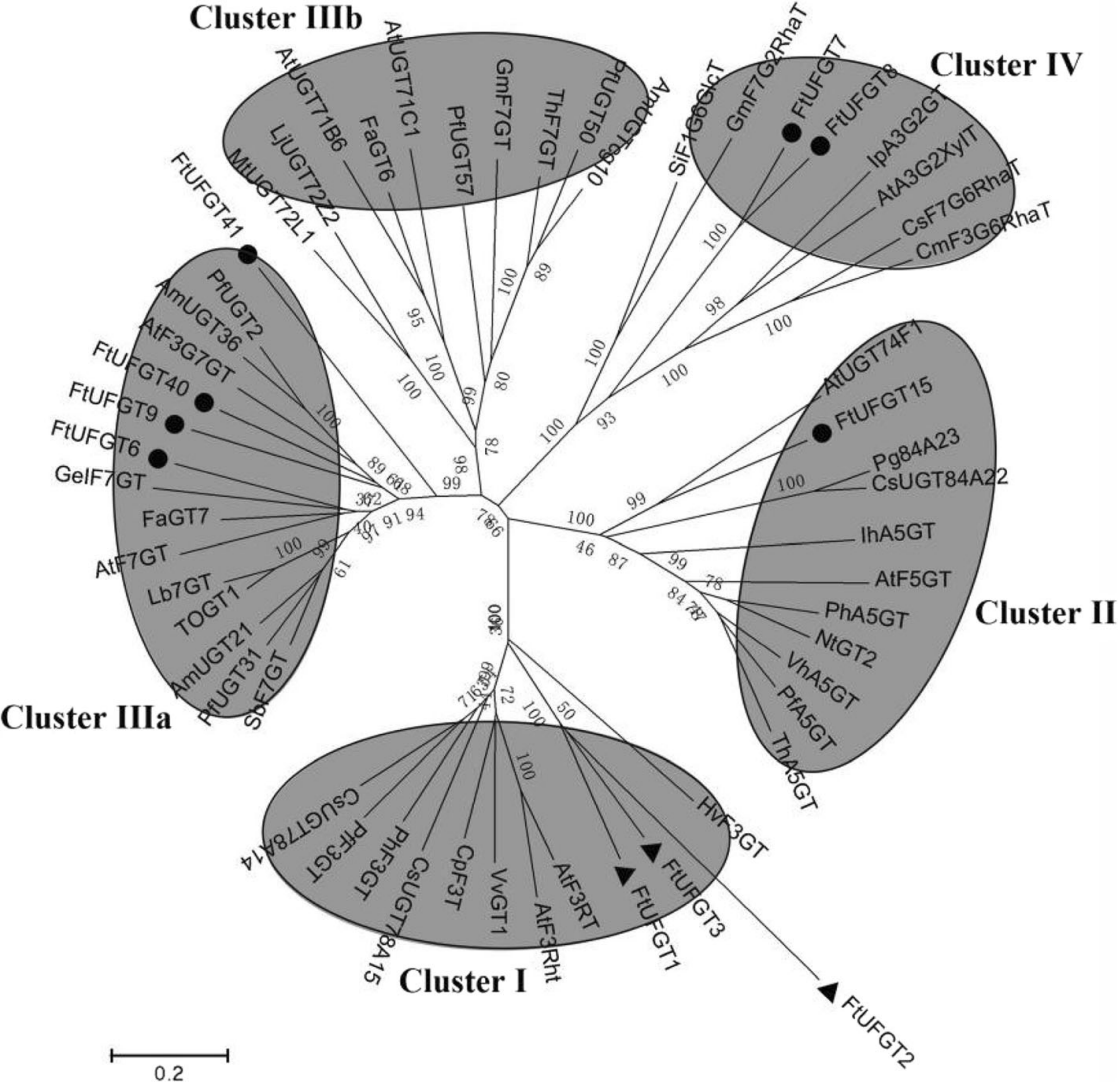
Phylogenetic analysis of selected plant GTs and putative tartary buckwheat UFGTs. Bar = 0.2 amino acid substitutions per site. Functional clusters (I, II, IIIa, IIIb and IV) of flavonoid UGTs are circled. Black point represent UFGTs in tartary buckwheat. Black triangle represent UFGTs have been studied in tartary buckwheat. The Genbank accession numbers for the sequences are shown in parentheses: AtF3RT (NP_197207); PfF3GT (BAA19659); PhF3GT (BAA89008); HvF3GT (CAA33729); VvGT1 (AAB81682); AtF3Rht (NP_564357); CpF3T (ACS15351); CsUGT78A14 (ALO19888); CsUGT78A15 (XP_028088706); VhA5GT (BAA36423); AtF5GT, AAM91686; PfA5GT, Q9ZR27; PhA5GT, BAA36421; ThA5GT, BAC54093; NtGT2, BAB88935; IhA5GT (Q767C8); AtUGT74F1 (NP_973682); Pg84A23 (ANN02875); CsUGT84A22 (ALO19890); TOGT1 (AAK28303); AtF7GT (AAL90934); GeIF7GT (BAC78438); SbF7GT (BAA83484); Lb7GT (BAG80536); FaGT7 (Q2V6J9); AmUGT21 (BAG31950); PfUGT31 (BAG31952); AmUGT36 (BAG16513); PfUGT2 (BAG31951); AtF3G7GT (Q9ZQ95); MtUGT72L1 (ACC38470); AtUGT71B6 (NP_188815); LjUGT72Z2 (AKK25344); FaGT6 (Q2V6K0); AtUGT71C1 (NP_180536); ThF7GT (BAH14961); PfUGT57 (BAG31949); PfUGT50 (BAG31948); AmUGTcg10 (BAG31945); IpA3G2GT (BAD95882); SiF1G6GlcT (BAF99027); GmF7G2RhaT (Q8GVE3.2); AtA3G2XyIT (NP_200217); CsF7G6RhaT (NP_001275829); CmF3G6RhaT (NP_001275524); FtUFGT1 (KX216512); FtUFGT2 (KX216513); FtUFGT3 (KX216514)

Multiple sequence analysis showed that the 7 UFGTs shared a conserved domain with the plant secondary product glycosyltransferase (PSPG) motif (Fig. 1) near their C-terminal domain, and the highly conserved amino acids were in positions 1 (W), 4 (Q), 8 (L), 10 (H), 12 (A/S), 14 (G), 16 (F/C), 19–24 (HC/SGW/FN/GS), 27 (E), 32 (G/N), 39 (P), 43 (E/D), and 44 (Q). This is consistent with the glycosyltransferases that are known to function in the biosynthesis of plant secondary metabolites [20]. The final glutamine (Q) residue within the PSPG motif is thought to confer specificity for UDP-glucose as the sugar donor [21]. Notably, all 7 UFGTs possess this Q, suggesting they may all use UDP-glucose as a sugar donor. The phylogenetic tree of putative FtUFGTs and *Arabidopsis* UDP glycosyltransferases indicated four clusters, which appear to be characterized by the specificity of the flavonoid glycosyltransferase activities (Fig. 2). Clusters I, II and III are characterized by flavonoid 3-O-glycosyltransferases, flavonoid 5-O-glycosyltransferases and flavonoid 7-O-glycosyltransferases, respectively. The results showed that six *FtUFGT* genes, including *FtUFGT6*, *FtUFGT7*, *FtUFGT8*, *FtUFGT9*, *FtUFGT40*, and *FtUFGT41*, were clustered into the UF7GT cluster, and *FtUFGT15* belonged to the UF5GT cluster. Additionally, *P. frutescens* and *P. hybrida* UF3GT did not cluster with *P. frutescens* and *P. hybrida* UF5GT, although they were derived from the same species. These results implied that the seven UDP glycosyltransferase clusters diverged before the speciation of monocot and dicot plants as reported by Imayama et al. [22] To further understand the relationship between these genes, we examined the location of the seven genes on the tartary buckwheat chromosomes. *FtUFGT8* and *FtUFGT15* are on chromosome Ft3, and the remainder is on different chromosomes. *FtUFGT6* is on chromosome Ft7, *FtUFGT7* is on chromosome Ft6, *FtUFGT40* is on chromosome Ft5, and *FtUFGT41* is on chromosome Ft2. However, we could not locate *FtUFGT9* on the tartary buckwheat genome, possibly because of the differences between different species.

### Expression of *FtUFGT* genes in different tissues

**Fig. 3:**
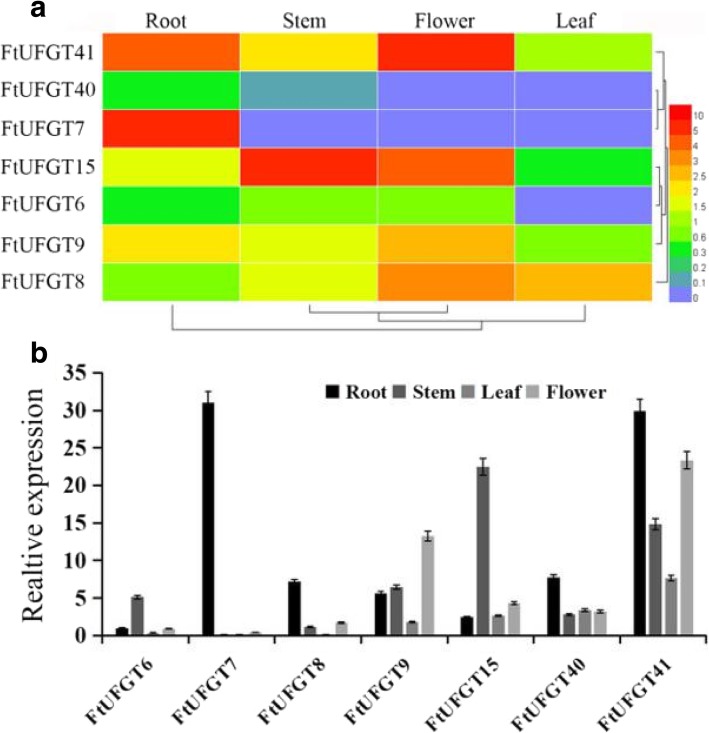
Tissue-specific expression of *FtUFGT* genes in flowering stages of tartary buckwheat. (**a**) Heat map of *FtUFGTs*. Each column represents one tissue, each line represents one gene which are displayed at the right. Depths of color in the blue and red rectangles reflect lower and higher *Z*-scores for mRNA accumulations. (**b**) Expression pattern of *FtUFGTs*. *FtH3* was used as a reference gene. The accumulation of *FtUFGT6* mRNA in the root was defined at “1”. Means were calculated from three repeats, and error bars reflect ±SD

Tissue-specific expression of genes is often associated with specific developmental and physiological functions. Therefore, we detected the expression levels of these *UFGT* genes in three tissues (root, stem, and leaf) at different growth stages (seedling stage, cotyledon stage, true leaf stage, full-leaf stage, and full-bloom stage) of tartary buckwheat by real-time quantitative PCR (qRT-PCR). Most genes showed different expression patterns in tartary buckwheat, whereas *FtUFGT15* and *FtUFGT41* presented a similar expression pattern (Additional file 3: Figure S3). After the seedling stage, the expression levels of these two genes were highest in stems, followed by leaves, and lowest in roots, implying that they may participate in similar biological pathways. By contrast, both *FtUFGT6*, *FtUFGT7*, and *FtUFGT40* have the highest expression in roots at different growth stages. Particularly, *FtUFGT7* was almost undetectable in the leaves and stems after the seedling stage, suggesting that *FtUFGT7* may be a root-specific gene. Additionally, the expression levels of *FtUFGT8* and *FtUFGT9* in the leaves were always at a relatively low level but showed opposite trends in roots and stems. At the same time, transcriptome data analysis and qRT-PCR were performed to determine the expression levels of these genes in flowering tartary buckwheat (Fig. 3a). The results indicated that gene expression did not change markedly compared with the early stage and remained consistent, maintaining almost the same tissue specificity in different growth stages of tartary buckwheat (Additional file 3: Figure S3).

### Isolation and sequence analysis of the *pFtUFGTs*

To further reveal the response of the *FtUFGT* genes to the external environment and predict the regulatory pathways they may be involved in, we cloned the promoters starting between 1567 and 1594 bp upstream of the ATG start codon (Additional file 4: Figure S4). Analysis of the *cis*-regulatory elements in the promoter showed that these elements were classified into two groups based on their responsive functions: stress-responsive and hormone-responsive elements (Additional file 1: Table S2). The stress-responsive elements mainly included light-responsive elements (e.g., Box 4, ATTAAT), low-temperature (e.g., LTR, CCGAAA), drought (e.g., MBS, CAACTG), and high-temperature responsive elements (e.g., HSE, AGAAAATTCG). The hormone-responsive elements included abscisic acid (ABA)-responsive elements (ABRE, ACGTG), an auxin-responsive element (e.g., TGA-element, AACGAC), and the MeJA-responsive element (e.g., CGTCA-motif, CGTCA). Furthermore, several other types of *cis*-acting elements were found in these promoter sequences, including many TATA boxes, CAAT boxes, and MYB binding sites. The results of the analysis showed that these promoters contain numerous photoresponsive elements, and we also found that *pFtUFGT8/15/41* contains more low-temperature response components than the other four promoters (Additional file 1: Table S1). It is well known that illumination directly affects the secondary metabolism of plants, and low temperature can induce the accumulation of anthocyanins by activating the expression of anthocyanin synthesis-related genes [23, 24]. Therefore, we speculate that *FtUFGT8/15/41* may be involved in the production of anthocyanin.

### *FtUFGT8/15* gene expression is correlated with anthocyanin accumulation after cold treatment

Based on the analysis result of *pFtUFGTs*, we carried out low-temperature treatment on the tartary buckwheat seedlings and explored the effects of low temperature on the synthesis of anthocyanins and expression of *FtUFGT* genes. We found that, compared with the control group, the anthocyanin content of tartary buckwheat increased significantly after cold stress, and there was a significant difference after 2 h (*P* < 0.01) (Additional file 5: Figure S5). The difference was greatest after 3 h, which was 1.72 fold that of the control group. Similar results have been reported in previous literature [18, 24].

To analyze the relationship between these *FtUFGT* genes and anthocyanin accumulation, the expression profiles of *FtUFGTs* in tartary buckwheat under cold treatment were analyzed by qRT-PCR. Overall, the seven *FtUFGT* genes showed different expression patterns under cold stress (Additional file 5: Figure S5). The expression of the five *FtUFGT* genes, *FtUFGT8*, *FtUFGT9*, *FtUFGT15*, *FtUFGT40*, and *FtUFGT41*, were clearly enhanced. Among them, the response of *FtUFGT9* and *FtUFGT41* was the most rapid, increasing significantly after 0.5 h of stress and remaining at a relatively high level thereafter. *FtUFGT8* and *FtUFGT15* expression did not change much in the early stage of stress, and they rose rapidly after 6 h and reached the maximum at 16 h, 10.88-fold and 24.36-fold of the control, respectively. However, the *FtUFGT7* gene showed downregulated expression. It remained unchanged within 0–2 h, significantly decreased after 3 h, and reached a minimum at 16 h, which was 0.27-fold that of the control.

### Expression of *FtUFGTs* and flavonoid accumulation in tartary buckwheat sprouts after light treatment

Light is one of the most important environmental factors affecting flavonoid biosynthesis in plants [25]. From the results of the UFGT promoter structure analysis, it was found that the promoter portion of these genes contained numerous photoresponsive elements. Hence, we analyzed the trend between the expression of *FtUFGT* genes and accumulation of flavonoids in tartary buckwheat under light conditions. The results showed that the accumulation of four flavonols under light conditions indicated different trends (Additional file 6: Figure S6). Among them, the content of rutin was not significantly different from the control within 3 h after treatment, significant differences occurred after 6 h of treatment, lasting 16 h. Additionally, the change in quercetin and kaempferol indicated similar trends. The trend of treatment for 3 h was similar to that of rutin, but the accumulation under light conditions was significantly lower than that under dark conditions after 6 h of treatment. However, the content of myricetin was higher under the dark conditions than in the light throughout the treatment.

Subsequently, the expression profiles of *FtUFGT*s in tartary buckwheat under light treatment were analyzed by qRT-PCR. Overall, the seven *FtUFGT* genes indicated different expression patterns under light stress (Additional file 7: Figure S7). The expression of the three *FtUFGT* genes, *FtUFGT6*, *FtUFGT15*, and *FtUFGT40*, was not significantly different before 3 h of treatment, but there was a significant change after 6 h, and light conditions obviously inhibited the expression of these three genes. Additionally, the expression level of *FtUFGT8* was decreased sharply after 0.5 h of treatment and reached the minimum value after 2 h, 0.504 times that of the control. Then, it rose rapidly and reached the maximum value after treatment for 16 h, 3.938 times that of the control group. *FtUFGT41* and *FtUFGT9* showed a trend of increasing first and then decreasing during the whole process. Overall, these seven genes all responded to light conditions, but the trends were somewhat different. It is speculated that they may play different roles in the flavonoid synthesis pathway.

### *FtUFGT8*, *FtUFGT15*, and *FtUFGT41* increase the anthocyanin content of transgenic plants

To further clarify the function of the seven selected *FtUFGT* genes in plants, transgenic *Arabidopsis thaliana* overexpressing *FtUFGT* genes were obtained by the floral dipping method. Eight resistant strains were selected in each plate and were found to be positive by RT-PCR. The results showed that the *FtUFGT* genes were expressed in all resistant seedlings but were not detected in wild type (WT) plants (Additional file 8: Figure S8). Thereafter, we selected three transgenic lines with higher *FtUFGT* gene expression levels among the T1 lines, and T3 homozygous plants were obtained for follow-up experiments.

**Fig. 4:**
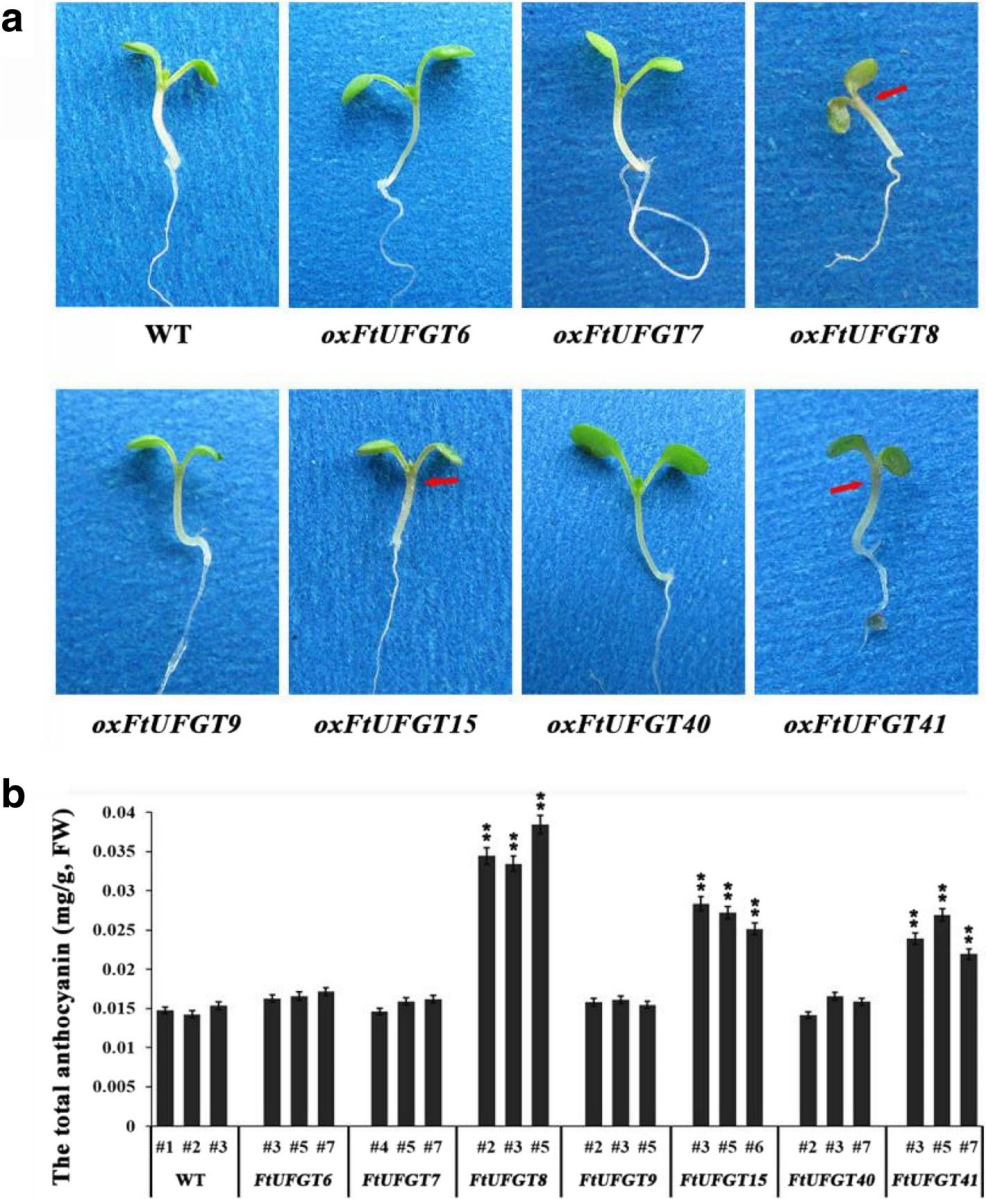
(**a**) Phenotypic of seedling transgenic plants and wild type. The seedlings of the transgenic plants and wild type were grown on 1/2 MS medium for 2 weeks. Red arrow represent where the color is deepened. (**b**) The total anthocyanin contents in transgenic plants and wild type. Each value represents the mean of three replicates, and error bars indicate standard deviations (±SD). * and ** represent significant differences between transgenic lines and WT at *P* < 0.05 and *P* < 0.01, respectively

When the transgenic and WT plants were grown on 1/2MS medium, the transgenic *oxFtUFGT8*, *oxFtUFGT15*, and ox*FtUFGT41* seedlings developed a slight purple color, indicative of anthocyanin accumulation, which was not present in the four other transgenic lines (Fig. 4a). Therefore, we speculated that *FtUFGT8*, *FtUFGT15*, and *FtUFGT41* may be involved in the synthesis of anthocyanins. After the plants grew to the flowering stage, their anthocyanin content was determined. Overexpression of the three genes *FtUFGT8*, *FtUFGT15*, and *FtUFGT41* significantly increased the anthocyanin content of the transgenic plants, which were 2.50-, 1.78-, and 1.66-fold the content of the control group, respectively (*P* < 0.01) (Fig. 4b).

### *FtUFGTs* affect the accumulation of major flavonols in transgenic Arabidopsis

**Fig. 5:**
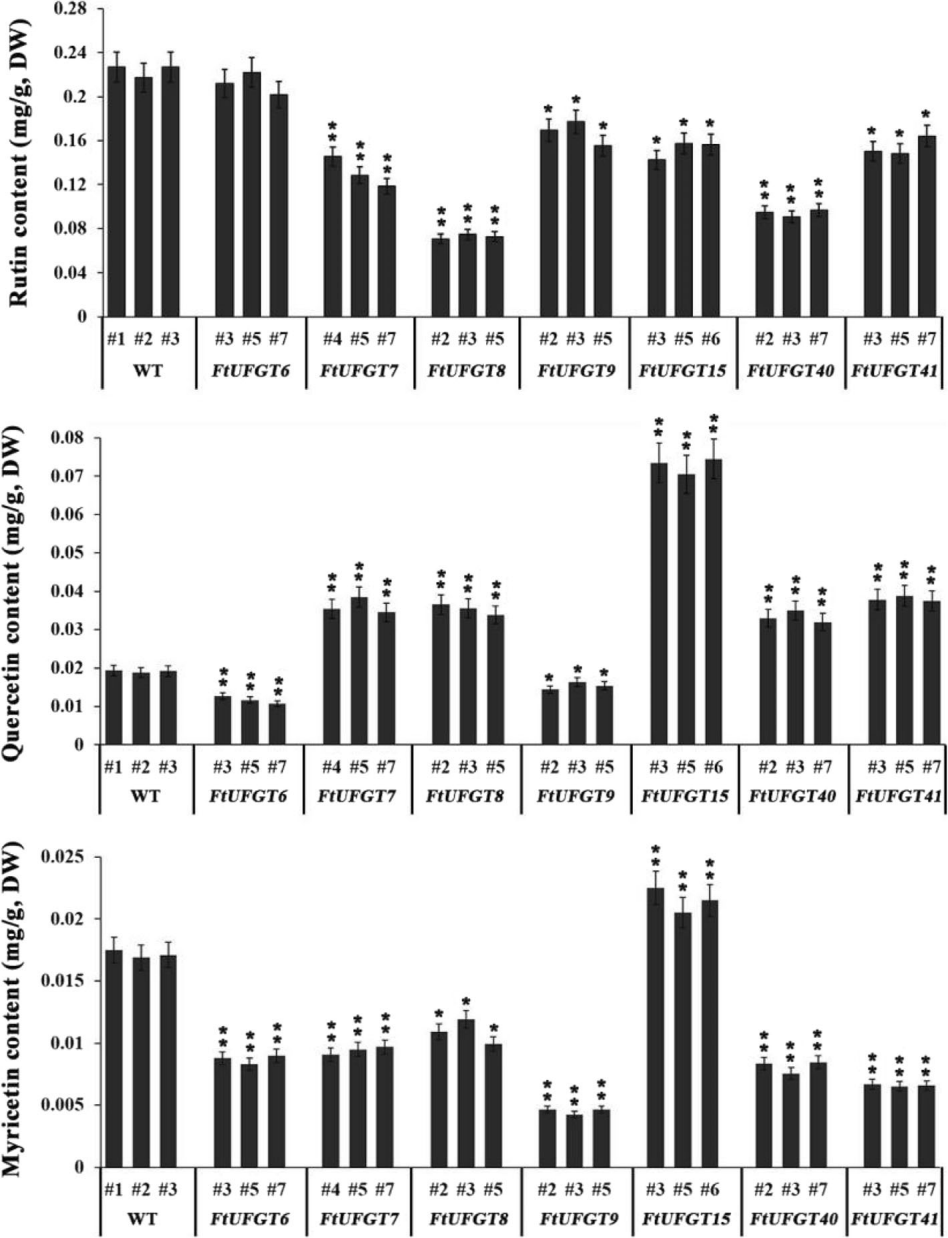
The main flavonoid contents (rutin, quercetin, and myricetin) in transgenic plants and wild type. Each value represents the mean of three replicates, and error bars indicate standard deviations (±SD). * and ** represent significant differences between transgenic lines and WT at *P* < 0.05 and *P* < 0.01, respectively

To clarify the effect of *FtUFGT* genes on flavonoid biosynthesis in transgenic plants, we tested the three main flavonols (rutin, quercetin, and myricetin) by high performance liquid chromatography (HPLC) (Fig. 5, Additional file 9: Figure S9). For all genes, except for *FtUFGT6*, overexpression resulted in a significant decrease in rutin content in the transgenic plants (*P* < 0.05). Among them, *FtUFGT8* transgenic plants showed the most significant reduction, at 0.35 fold that of wild type plants (*P* < 0.01). However, the effect of overexpression of genes on quercetin and rutin showed the opposite trend. Except for *FtUFGT6* and *FtUFGT9*, the overexpression of other genes, including *FtUFGT7*, *FtUFGT8*, *FtUFGT15*, *FtUFGT40*, and *FtUFGT41*, significantly increased the content of quercetin in the transgenic plants, by 2.05-, 1.94-, 3.89-, 1.83-, and 2.05-fold of the WT, respectively (*P* < 0.01).

### *FtUFGT6* and *FtUFGT15* affect the growth and development of transgenic plants

**Fig. 6:**
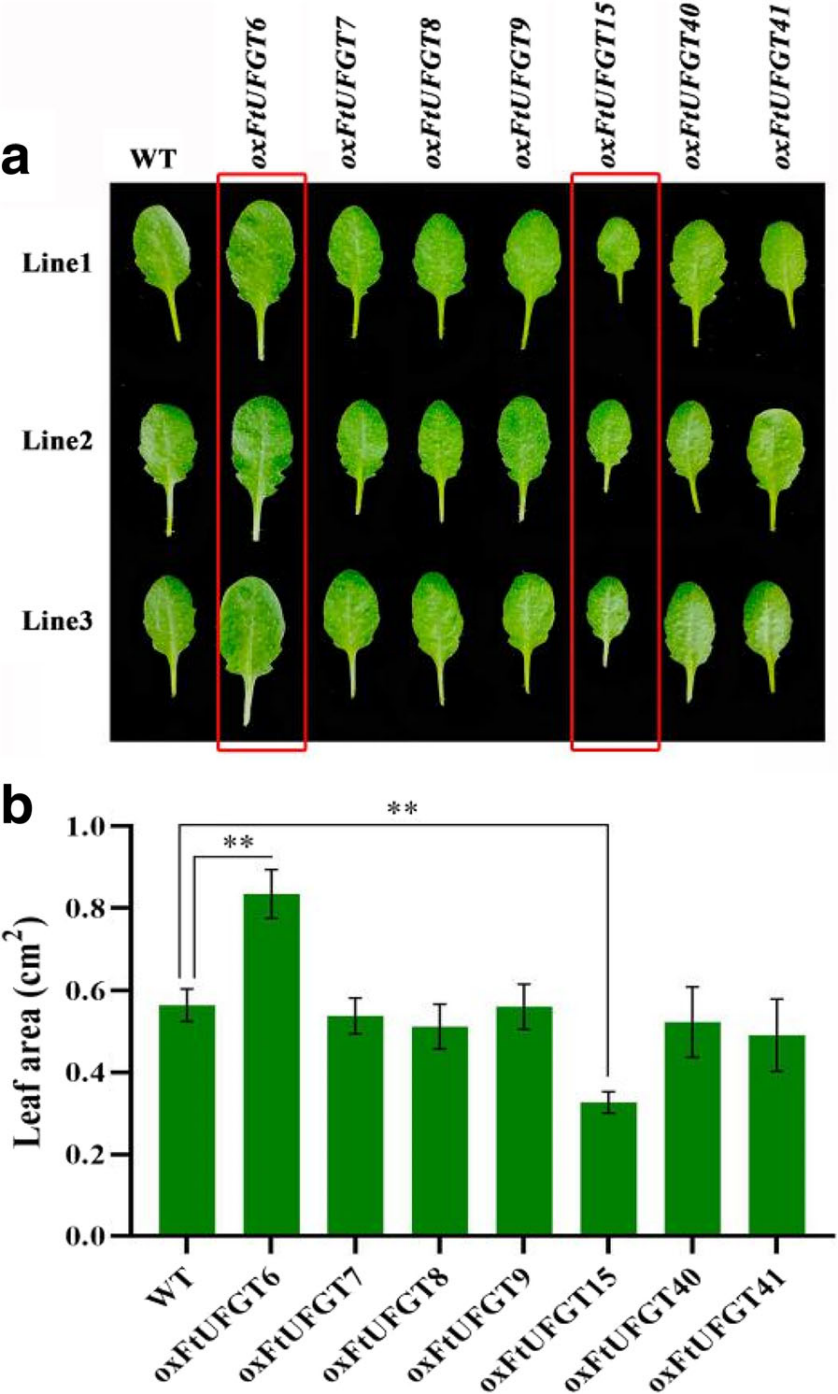
Leaf size of transgenic plants and wild type. (**a**) The seedlings of the transgenic plants and wild type were grown on soil for about 40 days. The transgenic plants of *FtUFGT6* and *FtUFGT15* are marked with a red frame. (**b**) Leaf area was quantified by Image J software. Each value represents the mean of three replicates, and error bars indicate standard deviations (±SD). ** indicate a significant difference from that of WT at *p* < 0.01

**Fig. 7:**
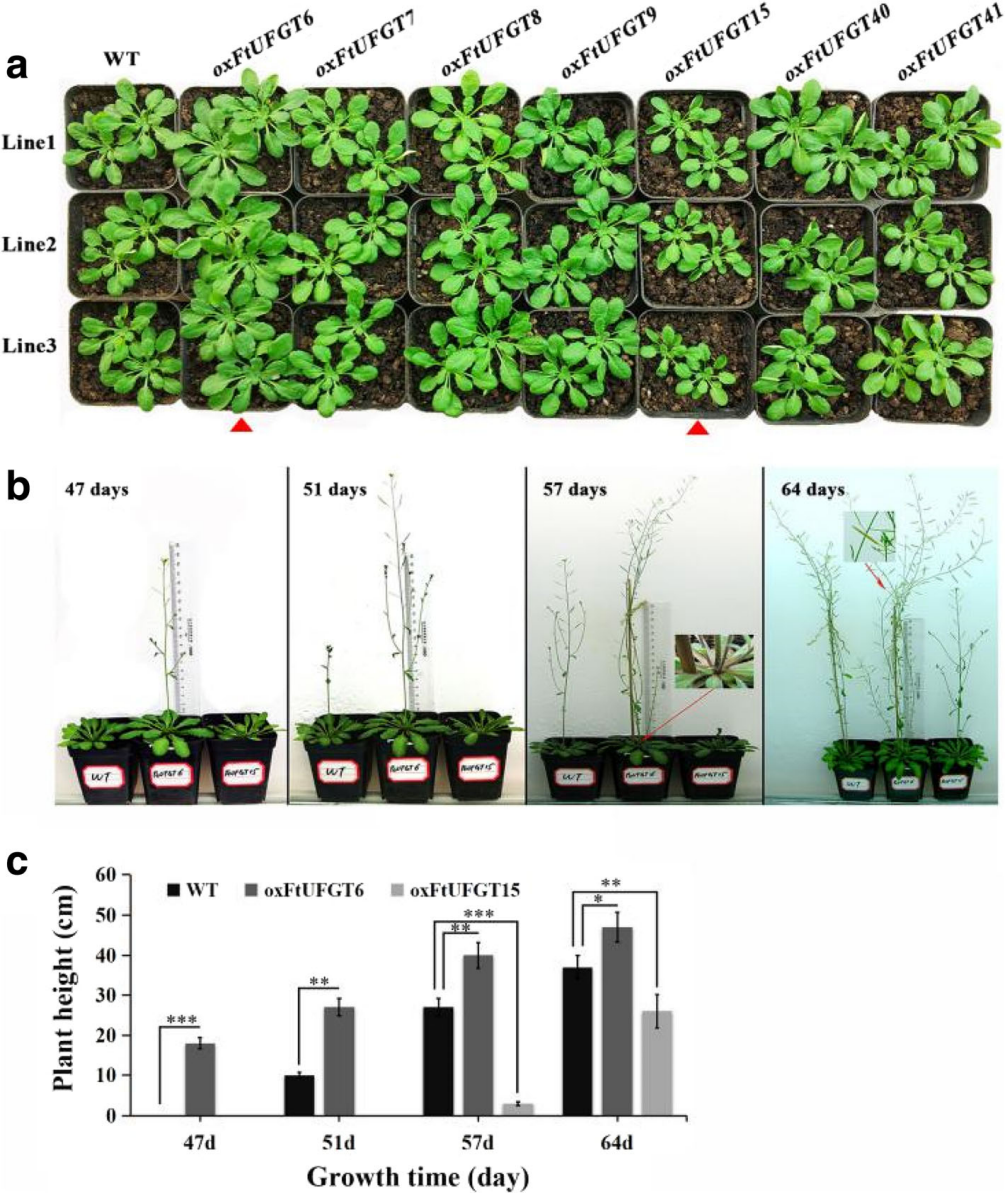
Phenotype of transgenic plants and wild type. (**a**) The rosette phenotype of 40-day old transgenic plants. Red arrow highlights *FtUFGT6* and *FtUFGT15* transgenic plants. (**b**) The phenotype of transgenic plants at four different growth stages (47-day, 51-day, 57-day, and 64-day). (**c**) The plant height of *FtUFGT6* and *FtUFGT15* transgenic plants at four different growth stages (47-day, 51-day, 57-day, and 64-day). Each value represents the mean of three replicates, and error bars indicate standard deviations (±SD). *, **, and *** indicate a significant difference from that of WT at *p* < 0.05, *p* < 0.01, *p* < 0.001, respectively

Unexpectedly, the overexpression of two of the FtUFGT genes affected the growth and development of transgenic plants. Leaf size of *oxFtUFGT6* and *oxFtUFGT15* plants showed greater differences than the WT and other transgenic plants when the same batch of transgenic plants were grown to approximately 40 days (Fig. 6a and b). The whole rosettes of the *oxFtUFGT6* plants were significantly larger than those of WT plants, whereas *oxFtUFGT15* showed the opposite trend (Fig. 7a). Divergent phenotypes were observed at different stages of development (Fig. 7b). At 47 days, *oxFtUFGT6* plants bolted ahead of WT and *oxFtUFGT15* plants. By day 51, the stems of *oxFtUFGT6* plants and WT plants had grown 27 and 10 cm, respectively. The stems of *oxFtUFGT15* plants grew 3 cm on day 57, at which time the plant heights of the other two plants reached 40 cm and 27 cm, respectively. Additionally, overexpression of *FtUFGT6* also increased the number of tillers and time for seed maturation of transgenic plants. The number of tillers overexpressing *FtUFGT6* reached 4 at 57 days, while the WT and *oxFtUFGT15* plants had only one until the end. By the 64th day, the seeds of plants that had overexpressed *FtUFGT6* had partially matured, while the other two were still dark green. Because AUXIN RESPONSE FACTORs ARF10 and ARF16 are the major auxin response factors in plants, the relative gene expression levels of *ARF10* and *ARF16* in these two transgenic plants were measured (Additional file 10: Figure S10). *ARF10* and *ARF16* expression levels increased in *oxFtUFGT6* plants, and the *ARF16* change was the most significant, reaching 4.01 times that of the control group. However, there was a different trend in *FtUFGT15* transgenic plants. *FtUFGT15* did not affect the gene expression of *ARF10* but markedly suppressed *ARF16* expression.

**Fig. 8:**
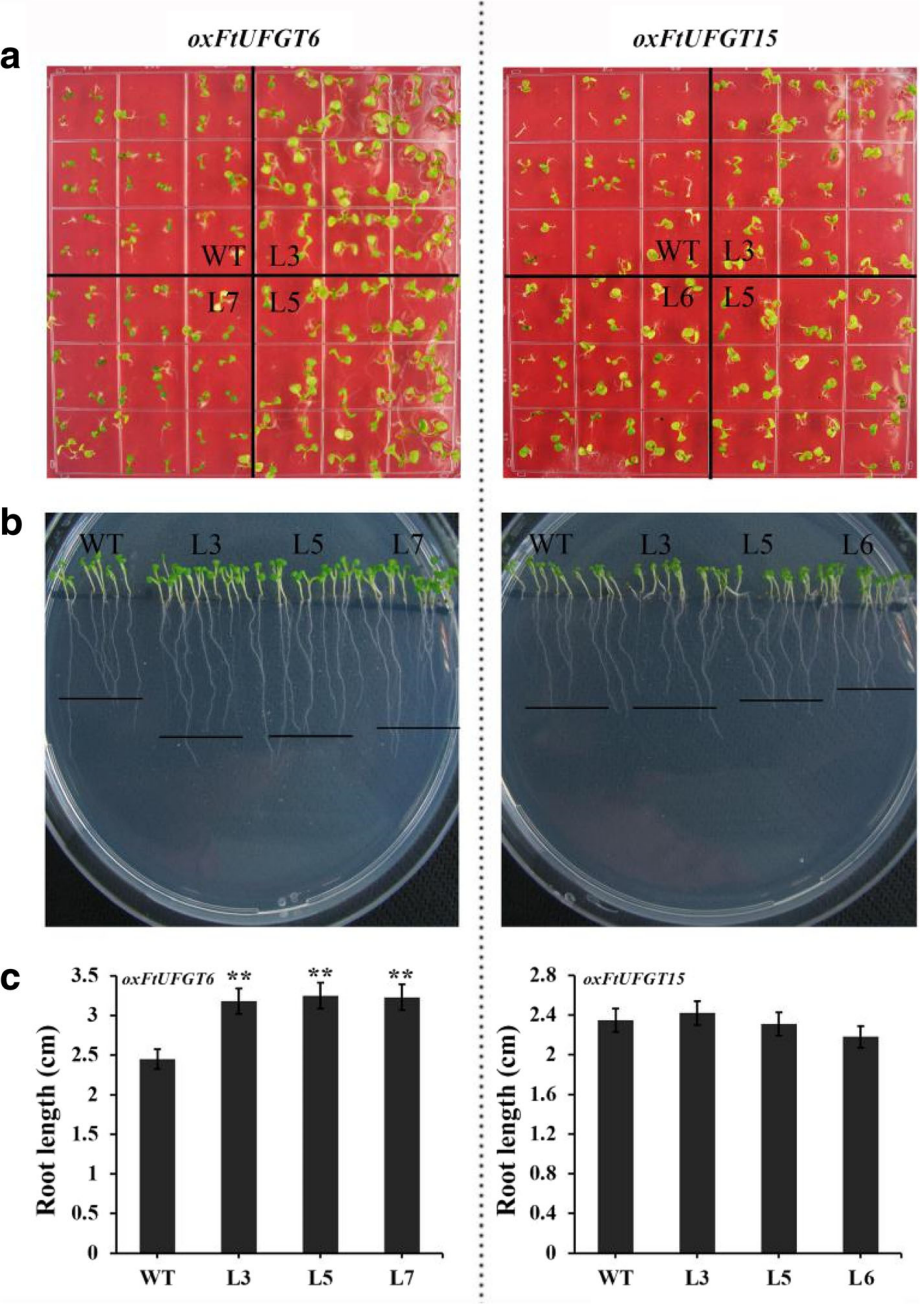
The growth of *oxFtUFGT6* and *oxFtUFGT15* transgenic plants at seedling stage. (**a**) The seedlings were grown on 1/2 MS medium for a week. (**b**) The root phenotype of transgenic plants on 1/2 MS medium grown for a week. (**c**) The root length of transgenic plants. Each value represents the mean of three replicates, and error bars indicate standard deviations (±SD). ** indicate a significant difference from that of WT at *p* < 0.01

Taken together, overexpression of *FtUFGT6/15* significantly affected the growth and development of transgenic plants. To investigate whether this effect also exists early in plant growth, we measured the developmental speed and root length of transgenic and WT seedlings on 1/2 MS medium. Overexpression of *FtUFGT6* significantly increased the early developmental speed of transgenic plants (Fig. 8a). When wild-type plants still have only two cotyledons, most of the transgenic plants of *FtUFGT6* have grown true leaves. Moreover, *oxFtUFGT6* plant root growth was increased (Fig. 8b and c). On the contrary, *oxFtUFGT15* seedling leaf and root growth did not significantly differ from wild type.

## Discussion

Glycosyltransferases are enzymes that catalyze the transfer of a glycosyl residue to an acceptor molecule. As of July 2019, 107 families of glycosyltransferases could be found in the Carbohydrate-Active Enzymes Database (CAZy) (http://www.cazy.org/GlycosylTransferases.html) according to sequence similarity, signature motifs, stereochemistry of the glucoside linkage, and target specificity [26, 27]. Glycosyltransferase acceptors include small compounds, including flavonoids, alkaloids, and hormones. Therefore, the functions of glycosyltransferases in plants are diverse. For instance, spontaneous mutations of the UDP-glucose: flavonoid 3-O-glucosyltransferase gene confers pale- and dull-colored flowers in the Japanese and common morning glories [28]. Overexpression of *CsUGT76F1* significantly increased the accumulation of kaempferol 7-O-glucoside, quercetin 7-O-glucoside, and quercetin 7-O-rhamnoside in transgenic plants [29]. In this study, the phylogenetic tree indicated selected UFGTs from tartary buckwheat were divided into different clusters depending on the glycosylation sites (Fig. 2), but these enzymes differ considerably in their substrate specificities. For example, among the enzymes in cluster IIIb, *MtUGT72L1* showed in vitro glucosyl-transferring activities toward epication 3′-O-glucosyltransferase, *GmF7GT* catalyzes the glucosylation of isoflavone at the 7-O-hydroxyl site, and *AtUGT71B6* may function as a hormone glucuronosyltransferase to transfer glucuronate onto ABA uridine diphosphate [30]. Such incongruence between the phylogenetic position and substrate specificities has been found in other UGTs, including grape *VLOGT2* and onion *UGT73G1* and onion *UGT73J1* [31, 32]. These results support the proposition that the functions and specificities of UGTs are perhaps not accurately determined based on their protein sequences alone [17]. Thus, the coupling of phylogenetic analyses with experimental analyses is generally regarded as the most efficient approach to identify UGT functions.

Anthocyanins are major compounds that contribute to the growth and flower coloring of plants. Stabilized anthocyanin is first produced by glycosylation at the 3-O-position via *UFGT*. In the case of orchids, the predominant anthocyanin is typically a cyanidin derivative that is modified by glycosylation [33]. These studies showed that glycosylation of anthocyanins is a prerequisite to flower color in plants. Previous studies have shown that cold stress induced anthocyanin accumulation in many plants, including quinoa [34], tartary buckwheat [24], and *Arabidopsis* [35]. In this study, anthocyanin accumulation was found in tartary buckwheat sprouts after cold treatment, similar to the response described by Li et al. [24] In a study of maize, nearly all of the anthocyanin synthesis genes were found to be upregulated in response to cold treatment [36, 37]. In our study, although similarly upregulated expression of the four *FtUFGTs* (*FtUFGT8*, *FtUFGT9*, *FtUFGT15*, and *FtUFGT40*) was observed in tartary buckwheat sprouts after cold treatment, *FtUFGT8* and *FtUFGT15* were expressed at the highest levels than others (Additional file 5: Figure S5). Similar results were demonstrated in red orange; that is, the transcript level of UFGT was increased by cold treatment [23]. Based on the results of specific activities and gene expression levels, we found that *FtUFGT8* and *FtUFGT15* were considered an important gene in the anthocyanin biosynthesis of tartary buckwheat.

It was reported that overexpression of some UGTs would increase or decrease the accumulation of flavonoids in plants. For example, overexpression of *CsUGT76F1* in tobacco increased the accumulation of quercetin 7-O-glucoside, quercetin 7-O-rhamnoside, and kaempferol 7-O-glucoside in transgenic plants [29]. The *LcUFGT1* overexpression tobacco had darker petals and pigmented filaments and calyxes resulting from higher anthocyanin accumulation than in control tobacco [14]. Additionally, mutating a single UGT gene also causes a decrease in the plant flavonoid content. Chen et al. [38] observed a significant decrease in the anthocyanin content of *Phalaenopsis* flowers following virus-induced gene silencing of *PeUFGT3*. These studies indicated that the method of identifying gene function by transgenic technology is very accurate. In this study, the ORF of seven *FtUFGTs* was transferred into *Arabidopsis thaliana*, and the results indicated that *FtUFGT8*, *FtUFGT15*, and *FtUFGT41* significantly increased total anthocyanin accumulation in transgenic plants (Fig. 4). Among them, *oxFtUFGT8* showed the highest accumulation, reaching 2.41 times that of the control group. The results indicated that these three genes might be directly involved in the synthesis of buckwheat anthocyanins. Surprisingly, overexpression of these genes not only affected the synthesis of anthocyanins but also affected the accumulation of the major flavonols (rutin, quercetin, and myricetin) in transgenic plants (Fig. 5). This result was not seen in previous studies of UFGTs. We speculate that there may be following reasons: one is that the protein encoded by the transferred gene may interact with other proteins, causing changes in the expression of key genes in other metabolic branches; the other is that the synthetic product of our introduced gene may act as a signaling molecule that regulates certain biological processes [39]. For example, at the epigenetic level, quercetin inhibits histone H1 and H2AX phosphorylation in plants, and catechol can bind to histones to regulate gene transcription [40]; at the transcriptional level, flavonoids can inhibit topoisomerase activity and regulate gene expression [39]. Besides, the change in the direction of metabolic flow may also be another important reason. It is well known that anthocyanin and flavonol synthesis pathways belong to this propane metabolic pathway, and there is also a competitive relationship to a certain extent [41]. When more substrates are used in anthocyanin synthesis, they are bound to affect the metabolic branches of flavonoids. This also provides a new perspective for future research in this area.

In addition to regulating secondary plant metabolism, UFGT can also affect plant growth and development [42]. This is due to the diversity of receptor molecules that are involved in glycosylation reactions in plants, such as secondary metabolites (flavonoids, anthranilate, monolignols, and caffeic acid), hormones (salicylic acid (SA), brassinosteroids, auxin, ABA, and cytokinin), and xenobiotics [43, 44]. When the glycosylated receptor is a hormone, glycosyltransferases will break the balance of hormone levels in the plant by modifying the hormone [45]. Because glycosylation affects aglycone properties such as bioactivity, solubility and transport, glycosylation is considered an important homeostatic mechanism for phytohormones. It was reported that several genes were involved in auxin glucosylation. For example, the main function of *UGT74E2* is to glycosylate indole-3-butyric acid (IBA). After inflorescence emergence, *UGT74E2OE* lines developed a clear shoot branching phenotype, and mature *UGT74E2OE* plants were also shorter in stature than wild-type plants [46]. Additionally, in these transgenic plants, not only were IBA-Glc concentrations increased but also free IBA levels were elevated and the conjugated IAA pattern was modified. This perturbed IBA and IAA homeostasis was associated with architectural changes, including increased shoot branching and an altered rosette shape [46]. Therefore, these studies provide solid evidence that auxin glycosylation plays important roles in regulating auxin homeostasis and plant development. Similarly, after overexpression of *FtUFGTs* in this study, the transgenic plants also exhibited similar growth status. Among them, *FtUFGT6*-overexpressing plants grew better at the seedling stage and the flowering time advanced, while *FtUFGT15*-overexpressing plants showed the opposite trend (Fig. 7). Therefore, we speculate that these two UFGT glycosylation-modified receptor molecules may also be plant hormones. After glycosylation, they break the hormone balance in the transgenic plants, thus affecting their respective growth and development. Additionally, glycosyltransferases show some temporal and spatial specificity in the regulation of plant development [42, 47]. Ectopic expression of *UGT75D1* resulted in smaller cotyledons than the wild type. However, the older plants eventually did not exhibit clearly different phenotypes than the wild type [46]. However, our study did not show similar results. *FtUFGT6/15* transgenic plants showed very significant differences from the seedling stage to the flowering stage than the wild type. Therefore, these studies suggested that *FtUFGT6/15* may be a very important player mediating the crosstalk between auxin homeostasis and plant growth.

## Conclusions

Seven *FtUFGTs* were isolated from tartary buckwheat. Anthocyanin accumulation in tartary buckwheat sprouts was rapidly induced in response to cold treatment and was correlated with the expression of the *FtUFGT8* and *FtUFGT15*. The transgenic *Arabidopsis* results showed that three *FtUFGTs*, *FtUFGT8*, *FtUFGT15*, and *FtUFGT41*, can significantly increase the accumulation of total anthocyanins in transgenic plants. Furthermore, *oxFtUFGT6* significantly increased the whole developmental period speed of transgenic plants. However, *FtUFGT15* showed opposite results at later growth stage. These results suggested that the biological function of *FtUFGT* genes in tartary buckwheat is diverse and can be further explored to improve flavonoid accumulation, plant growth and stress resistance.

## Methods

### Plant materials and treatments

Professor Anhu Wang of Xichang College gave the tartary buckwheat accessions “Xiqiao No. 2” used in this study; Since 2013, “Xiqiao No. 2” has been introduced into the Sichuan Agricultural University, Sichuan Province, China, and grown in experimental farm. Professor Yi Cai of Sichuan Agricultural University gave the *Arabidopsis thaliana* ecotype Columbia-0 (Col-0) used in this study. Tartary buckwheat (“Xi Qiao No.2”) was planted in a farm of Sichuan Agriculture University. The root, stem and leaf tissues of 5 different growth periods (germinating period, cotyledon period, true leaf period, mature period, and flowering stage) were collected to isolate and detect the expression profiles of *FtUFGTs*. For cold stress treatment, two-week-old seedlings were treated under 4 °C conditions; for light stress treatment, two groups of 7-day-old seedlings were treated under dark conditions for two days, after which one group was transferred to light conditions as an experimental group, and the other group was still cultured under dark conditions as a control group. The seedling samples were collected at 0, 0.5, 1, 2, 3, 6, 10, and 16 h and quick-frozen with liquid nitrogen and kept at − 80 °C for further study.

### Cloning and characterization of *FtUFGTs* DNA and cDNA sequences

The genomic DNA and RNA of tartary buckwheat were extracted using the Plant Genomic DNA Kit (TIANGEN, China) and RNAout Kit (TIANGEN, China), respectively. The cDNA was synthesized using a RevertAid First Strand cDNA Synthesis kit (Takara, Japan). The candidate *FtUFGT* genes were selected from the transcriptome database of tartary buckwheat (“Xi Qiao No.2”) constructed in our laboratory [19]. According to the obtained unigenes, the specific primers of seven *FtUFGT* candidate genes were designed using Primer 5, and the DNA sequences and cDNA sequences of these genes were amplified. The amino acid sequence alignments and phylogenetic tree were constructed using ClustalX and MEGA5, respectively. All the primers are listed in Additional file 11: Table S2.

### Overexpression FtUFGTs in *Arabidopsis*

*FtUFGT* genes were amplified from tartary buckwheat cDNA, which was inserted into the plant expression vector pCHF3. The recombinant vector pCHF3-35S-*FtUFGTs* was transformed into *Arabidopsis thaliana* Col-0 through the agrobacterium GV3101 [48]. After harvesting the T1 generation, the seeds were screened using 1/2 MS medium containing Kan (50 mg/L). After 2 weeks, the screened positive plants were transferred to the flower pots and were placed in an artificial climate chamber. Thereafter, the resistant seedlings were identified by RT-PCR. Three strains with the highest expression levels were selected for subsequent experiments.

### Determination of anthocyanin and flavonols in transgenic *Arabidopsis*

All fresh materials were collected, including transgenic *Arabidopsis* and tartary buckwheat, and the total anthocyanin content was extracted from various materials according to a previously reported method [49]. Next, 200 mg of samples were fully grind with liquid nitrogen, and then 1 ml of acidic methanol (1% HCl, v/v) was added. The samples were moderately shaken for 18 h at 25 °C and 100 rpm/min for extraction. Next, 500 μL of the supernatant was taken after 15 min of 16, 800×g centrifugation, and then an equal volume of deionized water and 300 μL of chloroform were added. After 5 min of 8000×g centrifugation, the content of anthocyanin was determined by taking the supernatant. The anthocyanin content was quantified using the following equation: Q_Anthocyanins_ = (A_530_ – 0.25 × A_657_) × M^−1^.

### Quantitative analysis by HPLC analysis

Samples (dry weight) were quick frozen with liquid nitrogen, and then 200 mg was weighed and added to 5 ml of methanol, followed by incubation for 1 h at 60 °C. After 10 min at 12000 rpm, the supernatant was filtered using a 0.45-μm organic phase filter. The flavonols were analyzed by HPLC using a C18 column (250 mm × 4.6 mm, 5 μm) at 30 °C as described previously. Standard products included rutin, quercetin, kaempferol, and myricetin, and the concentration of flavonols in the samples was calculated using a standard curve.

### qRT-PCR

The expression profiles of *FtUFGTs* in tartary buckwheat and transgenic *Arabidopsis* were detected by qRT-PCR. Each reaction included 7.5 μL of SYBR Green II Mix, 1 μL of cDNA template, 1 μL of primers, and 5.5 μL of double-distilled water. The PCR program was as follows: 95 °C for 3 min, 39 cycles of 95 °C for 5 s and 60 °C for 30 s. *FtH3* and *β-actin* served as reference genes in tartary buckwheat and *Arabidopsis*, respectively. The data were evaluated using the 2^−ΔΔCT^ method [50].

### Cloning and analysis of promoters

The promoters of the *FtUFGT* genes were selected from the tartary buckwheat genome database [51]. Specific primers of these promoters were designed using Primer 5.0 software, and the promoter sequences were amplified using tartary buckwheat genomic DNA as the template. The PCR products were subcloned into pMD™19-T and sequenced. The components of the *FtUFGT* gene promoter sequenced were analyzed and predicted using the promoter analysis database PLANTCARE (http://bioinformatics.psb.ugent.be/webtools/plantcare/html/) and plant cis-acting element analysis database PLACE (http://www.dna.affrc.go.jp/PLACE/). The promoter core analysis database Promoters (https://bip.weizmann.ac.il/toolbox/seq_analysis/promoters.html#databases) was used to predict the transcriptional starting site of *FtUFGTs*.

### Statistical analysis

To determine significant differences among the data, Student’s t test was conducted using SPSS 16.0.

## Additional files

Additional file 1: **Figure S1.** Phylogenetic tree showing clustering of 39 FtUFGT family members from *Fagopyrum tataricum*. The phylogenetic tree was constructed in MEGA5.0 using Neighbor-Joining and parsimony analytical methods. It contained 17 clustered groups, including groups of A, B, C, D, E, F, G, H, I, J, K, L, M, N, O, P, and Q. The Genbank accession numbers for the sequences are shown in parentheses: AtUGT79B1 (OAO90958); AtUGT89B1 (OAP14423); AtUGT89C1 (NP_563756); AtUGT90A1 (Q9ZVX4); AtUGT73B2 (XP_020875283); AtUGT73B3 (OAO99384); AtUGT73B4 (NP_179151); AtUGT73C1 (NP_181213); AtUGT73C5 (OAP09184); AtUGT72B1 (OAP00532); AtUGT72E2 (OAO95244); AtUGT72E3 (NP_198003); AtUGT78D1 (OAP13716); AtUGT78D2 (NP_197207); AtUGT85A1 (OAP13723); AtUGT76C1 (OAO89564); AtUGT76C2 (OAO93987); AtUGT76B1 (OAP05179); AtUGT83A1 (Q9SGA8); AtUGT87A1 (O64732); AtUGT86A1 (Q9SJL0); AtUGT84A3 (OAP00592); AtUGT84A4 (OAO98847); AtUGT84A2 (NP_188793); AtUGT75B1 (OAP16927); AtUGT75B2 (NP_172044); AtUGT75C1 (AAL69494); AtUGT74D1 (AAM61249); AtUGT74F1 (NP_181912); AtUGT92A1 (Q9LXV0); AtUGT82A1 (Q9LHJ2); GRMZM2G075387 (XP_008670630); GRMZM5G834303 (ACG33743); GRMZM2G082037 (ACF85065). (DOCX 110 kb)

Additional file 2: **Figure S2.** Genomic structures of seven FtUFGT genes from tartary buckwheat. Exons and introns are shown in boxes and lines, respectively. The numbers at the left and right side indicate the position of the translation start codon and stop codon, respectively. The numbers at the down side indicate the position of the splice junction site. (DOCX 36 kb)

Additional file 3: **Figure S3.** Tissue-specific expression and anthocyanin content of *FtUFGT* genes in different developmental stages of tartary buckwheat. SS, CS, TLS, FS represent seedling stage, cotyledon stage, true leaf stage and full-leaf stage of tartary buckwheat, respectively. **(A)** The expression pattern of *FtUFGTs*. *FtH3* was used as a reference gene. The accumulation of *FtUFGTs* mRNA in SS stage was defined at “1”. Means were calculated from three repeats; **(B)** The total anthocyanin contents in transgenic plants and wild type. Each value represents the mean of three replicates, and error bars indicate standard deviations (±SD). (DOCX 73 kb)

Additional file 4: **Figure S4.** The electropherogram of *FtUFGT.*promoters. (DOCX 42 kb)

Additional file 5: **Figure S5. (A)** Expression profiles of *FtUFGTs* after 4 °C treatment in tartary buckwheat seedlings were analyzed by qRT-PCR. The expression levels at 0 h (no treated) were set to “1” using the 2^−ΔΔCT^ method. Means were calculated from three repeats; **(B)** The total anthocyanin contents in tartary buckwheat seedlings under 4 °C treatment. Each value represents the mean of three replicates, and error bars indicate standard deviations (±SD). (DOCX 83 kb)

Additional file 6: **Figure S6.** Content of four kinds of flavonoids in tartary buckwheat seedlings under light treatment. Each value is the mean of 3 replicates, and error bars indicate standard deviations. (DOCX 65 kb)

Additional file 7: **Figure S7.** Expression profiles of *FtUFGTs* after light treatment in tartary buckwheat seedlings were analyzed by qRT-PCR. The expression levels at 0 h (no treated) were set to “1” using the 2^−ΔΔCT^ method. Means were calculated from three repeats. (DOCX 73 kb)

Additional file 8: **Figure S8.** Molecular analyses of the *FtUFGTs*-overexpressing Arabidopsis. Expression analysis of the *FtUFGTs* genes in transgenic plants and wild type. The Arabidopsis *Ataction* gene was used as an internal control. Data are presented as mean ± SD (*n* = 3). (DOCX 59 kb)

Additional file 9: **Figure S9.** HPLC chromatograph of flavonoids from standard samples **(A)**, wild type **(B)**, *oxFtUFGT8* plants **(C)**, *oxFtUFGT9* plants **(D)**, and *oxFtUFGT15* plants **(E)**; **a**-**d** represents rutin, myricetin, quercetin, and kaempferol, respectively; The number above the arrow indicates the peak area at different eluting time. (DOCX 76 kb)

Additional file 10: **Figure S10.** Expression analysis of the *ARF10* and *ARF16* genes in *FtUFGT6* and *FtUFGT15* transgenic plants.The accumulation of mRNA in wild type was defined at “1”. Means were calculated from three repeats. ***indicate a significant difference from that of WT at *p* < 0.001. (DOCX 41 kb)

Additional file 11: **Table S1.** Cis-element of *FtUFGTs* promoter function calculate. (XLSX 18 kb)

Additional file 12: **Table S2.** Primers used in this study. (DOCX 15 kb)

## Data Availability

Data supporting the results can be found in Additional files and any other datasets used and/or analyzed during the current study is available from the corresponding author on reasonable request.

## Abbreviations

ABA: Abscisic acid
ARF: Auxin response factor
CS: Cotyledon stage
FS: Full-leaf stage
GTs: Glycosyltransferases
HCl: Hydrochloric acid
HPLC: High performance liquid chromatography
IAA: Indole-3-acetic acid
IBA: Indole-3-butyric acid
MS: Murashige & Skoog
*ox*: overexpression
PCR: Polymerase chain reaction
PSPG: Plant secondary product glycosyltransferase
SA: Salicylic acid
SS: Seedling stage
TLS: True leaf stage
UDP: Uridine diphosphate
UFGT: UDP-glucose flavonoid glycosyltransferase
UV-B: Ultraviolet-B
WT: Wild type
