# Supplementary figures and images for: Diverse biological effects of glycosyltransferase genes from Tartary buckwheat
Source: BMC Plant Biol. 2019 Aug 5;19:339. doi: 10.1186/s12870-019-1955-z (PMC6683379; doi:10.1186/s12870-019-1955-z)

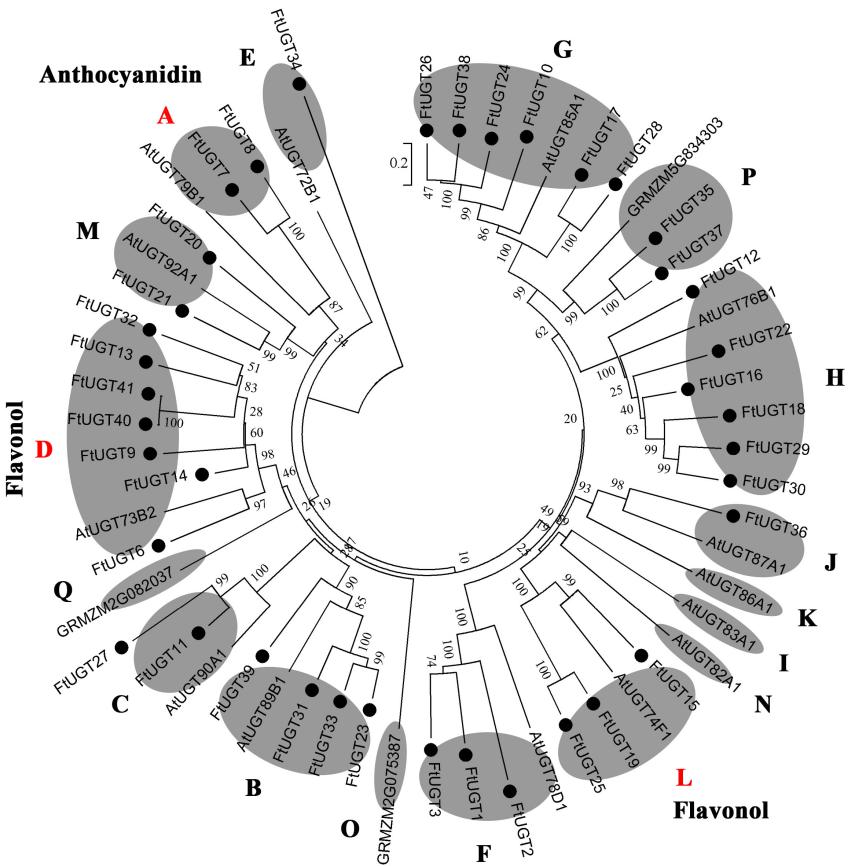

Supplement: Supplementary file 1 — Figure S1. Phylogenetic tree showing clustering of 39 FtUFGT family members from Fagopyrum tataricum. The phylogenetic tree was constructed in MEGA5.0 using Neighbor-Joining and parsimony analytical methods. It contained 17 clustered groups, including groups of A, B, C, D, E, F, G, H, I, J, K, L, M, N, O, P, and Q. The Genbank accession numbers for the sequences are shown in parentheses: AtUGT79B1 (OAO90958); AtUGT89B1 (OAP14423); AtUGT89C1 (NP_563756); AtUGT90A1 (Q9ZVX4); AtUGT73B2 (XP_020875283); AtUGT73B3 (OAO99384); AtUGT73B4 (NP_179151); AtUGT73C1 (NP_181213); AtUGT73C5 (OAP09184); AtUGT72B1 (OAP00532); AtUGT72E2 (OAO95244); AtUGT72E3 (NP_198003); AtUGT78D1 (OAP13716); AtUGT78D2 (NP_197207); AtUGT85A1 (OAP13723); AtUGT76C1 (OAO89564); AtUGT76C2 (OAO93987); AtUGT76B1 (OAP05179); AtUGT83A1 (Q9SGA8); AtUGT87A1 (O64732); AtUGT86A1 (Q9SJL0); AtUGT84A3 (OAP00592); AtUGT84A4 (OAO98847); AtUGT84A2 (NP_188793); AtUGT75B1 (OAP16927); AtUGT75B2 (NP_172044); AtUGT75C1 (AAL69494); AtUGT74D1 (AAM61249); AtUGT74F1 (NP_181912); AtUGT92A1 (Q9LXV0); AtUGT82A1 (Q9LHJ2); GRMZM2G075387 (XP_008670630); GRMZM5G834303 (ACG33743); GRMZM2G082037 (ACF85065). (DOCX 110 kb) [file 12870_2019_1955_MOESM1_ESM.docx]

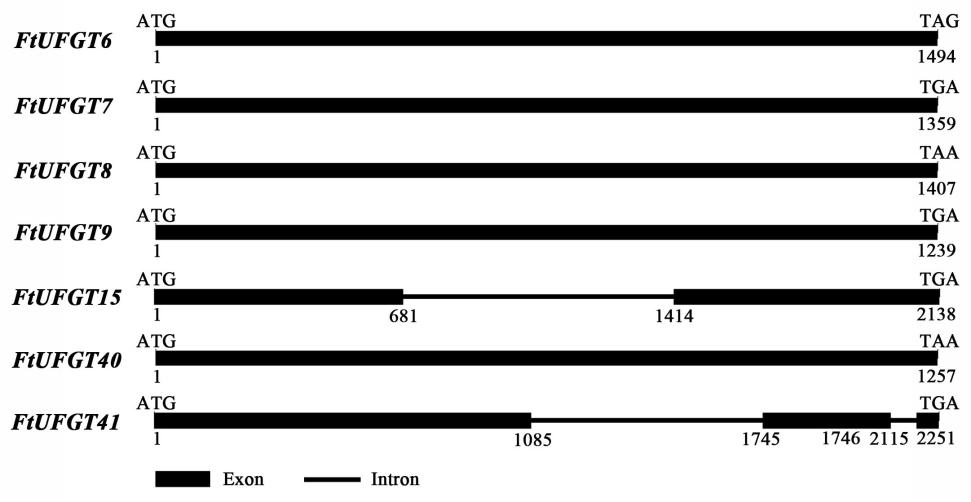

Supplement: Supplementary file 2 — Figure S2. Genomic structures of seven FtUFGT genes from tartary buckwheat. Exons and introns are shown in boxes and lines, respectively. The numbers at the left and right side indicate the position of the translation start codon and stop codon, respectively. The numbers at the down side indicate the position of the splice junction site. (DOCX 36 kb) [file 12870_2019_1955_MOESM2_ESM.docx]

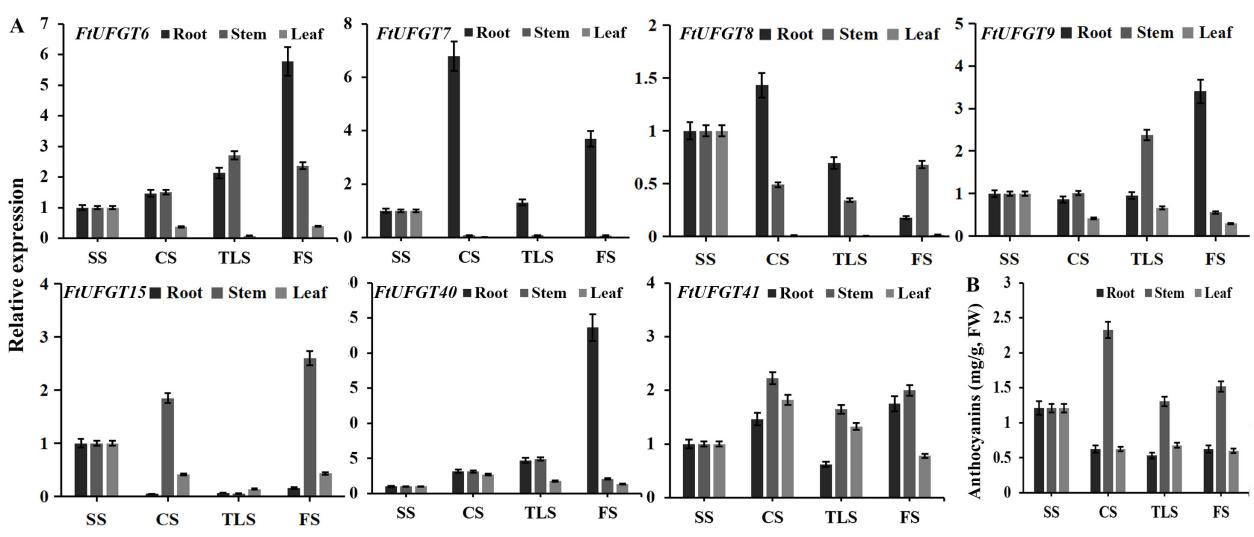

Supplement: Supplementary file 3 — Figure S3. Tissue-specific expression and anthocyanin content of FtUFGT genes in different developmental stages of tartary buckwheat. SS, CS, TLS, FS represent seedling stage, cotyledon stage, true leaf stage and full-leaf stage of tartary buckwheat, respectively. (A) The expression pattern of FtUFGTs. FtH3 was used as a reference gene. The accumulation of FtUFGTs mRNA in SS stage was defined at “1”. Means were calculated from three repeats; (B) The total anthocyanin contents in transgenic plants and wild type. Each value represents the mean of three replicates, and error bars indicate standard deviations (±SD). (DOCX 73 kb) [file 12870_2019_1955_MOESM3_ESM.docx]

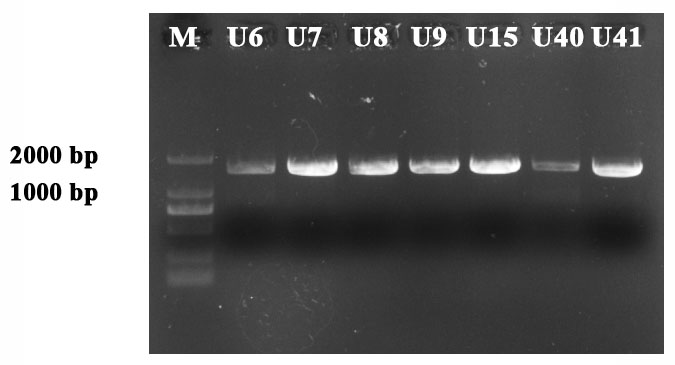

Supplement: Supplementary file 4 — Figure S4. The electropherogram of FtUFGT.promoters. (DOCX 42 kb) [file 12870_2019_1955_MOESM4_ESM.docx]

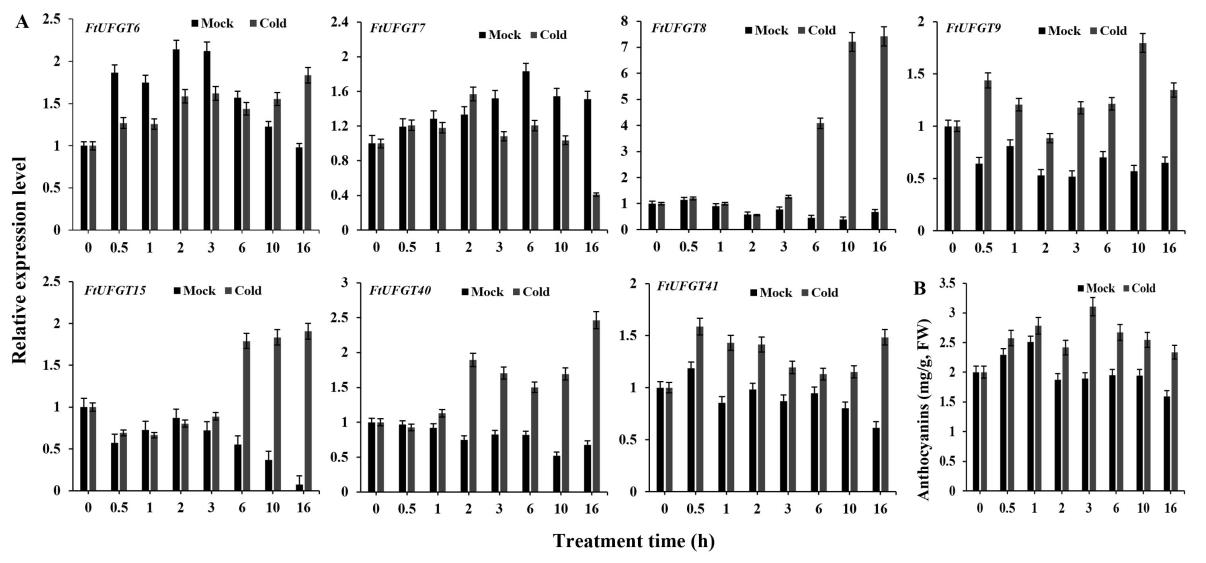

Supplement: Supplementary file 5 — Figure S5. (A) Expression profiles of FtUFGTs after 4 °C treatment in tartary buckwheat seedlings were analyzed by qRT-PCR. The expression levels at 0 h (no treated) were set to “1” using the 2−ΔΔCT method. Means were calculated from three repeats; (B) The total anthocyanin contents in tartary buckwheat seedlings under 4 °C treatment. Each value represents the mean of three replicates, and error bars indicate standard deviations (±SD). (DOCX 83 kb) [file 12870_2019_1955_MOESM5_ESM.docx]

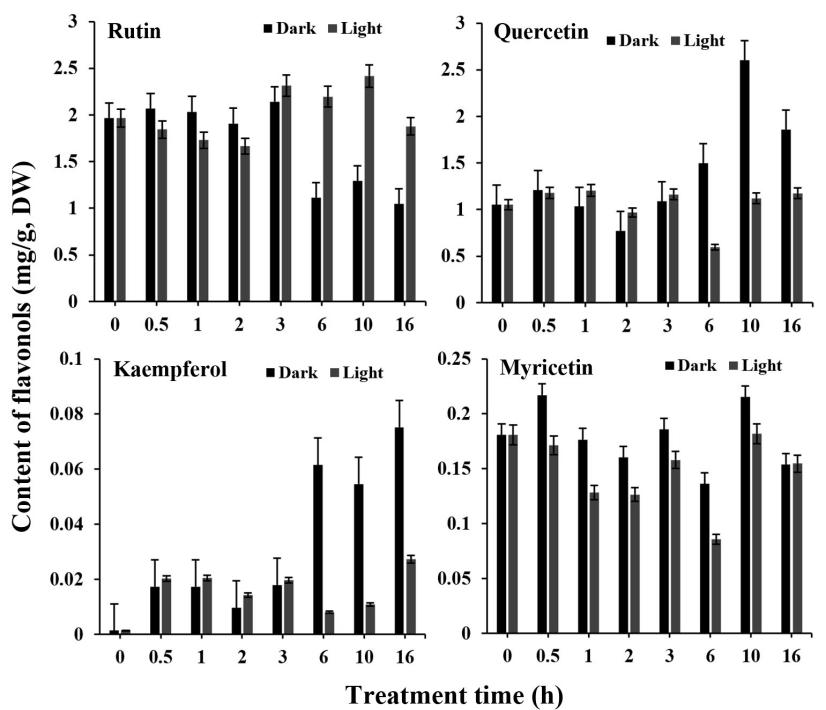

Supplement: Supplementary file 6 — Figure S6. Content of four kinds of flavonoids in tartary buckwheat seedlings under light treatment. Each value is the mean of 3 replicates, and error bars indicate standard deviations. (DOCX 65 kb) [file 12870_2019_1955_MOESM6_ESM.docx]

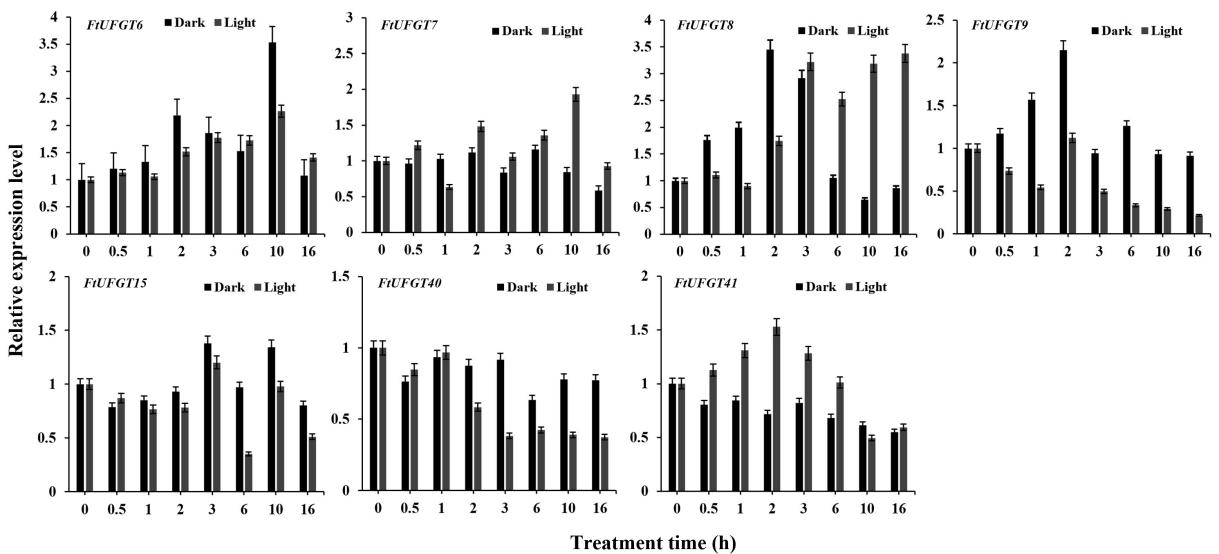

Supplement: Supplementary file 7 — Figure S7. Expression profiles of FtUFGTs after light treatment in tartary buckwheat seedlings were analyzed by qRT-PCR. The expression levels at 0 h (no treated) were set to “1” using the 2−ΔΔCT method. Means were calculated from three repeats. (DOCX 73 kb) [file 12870_2019_1955_MOESM7_ESM.docx]

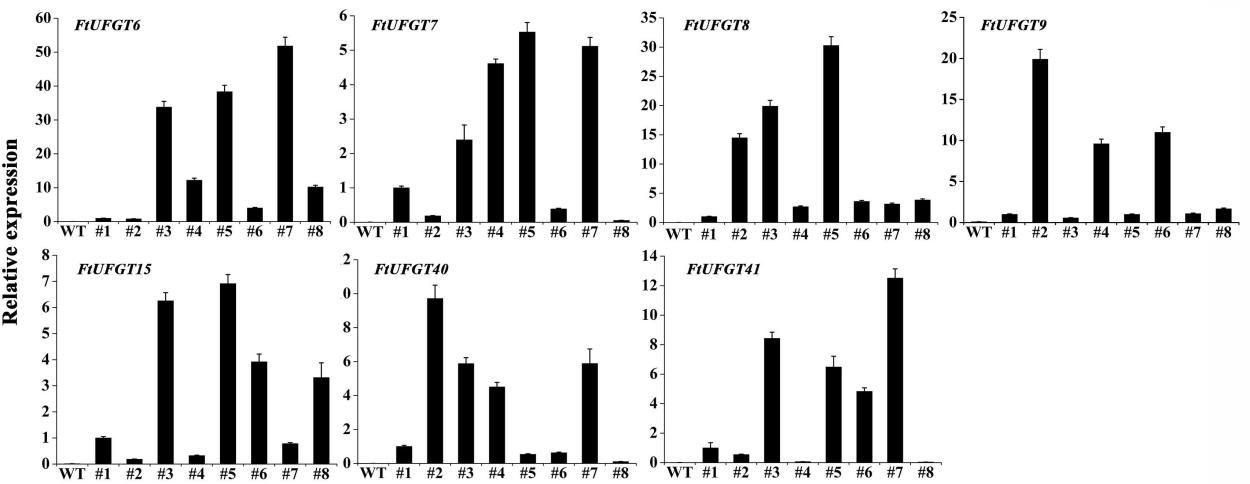

Supplement: Supplementary file 8 — Figure S8. Molecular analyses of the FtUFGTs-overexpressing Arabidopsis. Expression analysis of the FtUFGTs genes in transgenic plants and wild type. The Arabidopsis Ataction gene was used as an internal control. Data are presented as mean ± SD (n = 3). (DOCX 59 kb) [file 12870_2019_1955_MOESM8_ESM.docx]

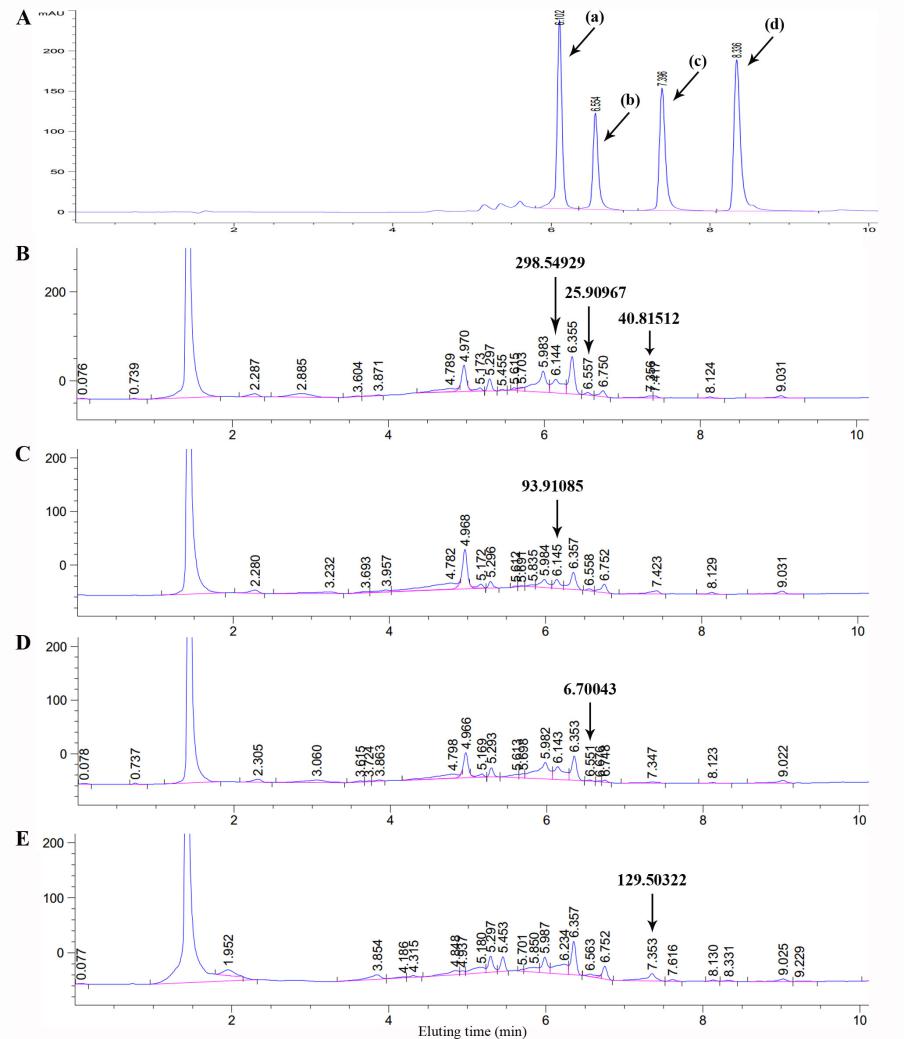

Supplement: Supplementary file 9 — Figure S9. HPLC chromatograph of flavonoids from standard samples (A), wild type (B), oxFtUFGT8 plants (C), oxFtUFGT9 plants (D), and oxFtUFGT15 plants (E); a-d represents rutin, myricetin, quercetin, and kaempferol, respectively; The number above the arrow indicates the peak area at different eluting time. (DOCX 76 kb) [file 12870_2019_1955_MOESM9_ESM.docx]

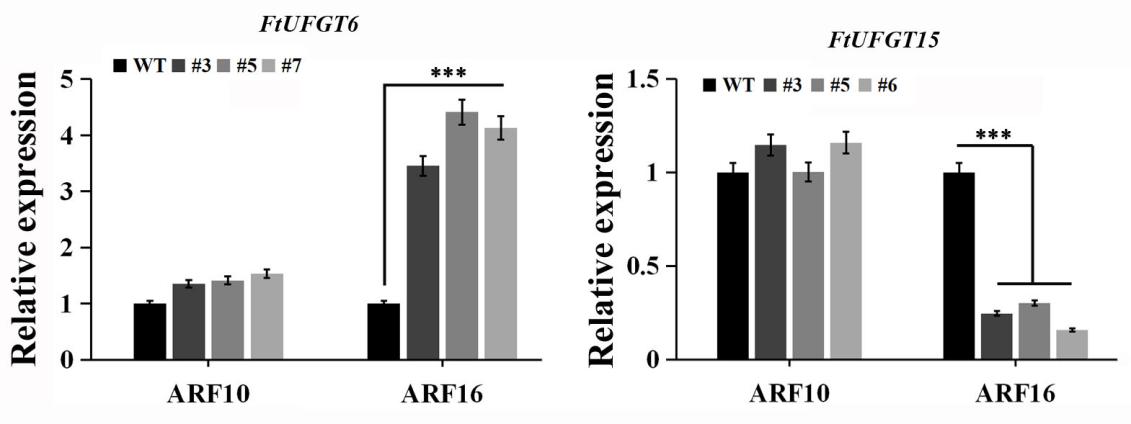

Supplement: Supplementary file 10 — Figure S10. Expression analysis of the ARF10 and ARF16 genes in FtUFGT6 and FtUFGT15 transgenic plants.The accumulation of mRNA in wild type was defined at “1”. Means were calculated from three repeats. ***indicate a significant difference from that of WT at p < 0.001. (DOCX 41 kb) [file 12870_2019_1955_MOESM10_ESM.docx]
