# Supplementary material for: Diverse biological effects of glycosyltransferase genes from Tartary buckwheat
Source: BMC Plant Biol. 2019 Aug 5;19:339. doi: 10.1186/s12870-019-1955-z (PMC6683379; doi:10.1186/s12870-019-1955-z)
Supplement: Supplementary file 12 — Table S2. Primers used in this study. (DOCX 15 kb) [file 12870_2019_1955_MOESM12_ESM.docx]

| **Primer name** | **Primer sequence (5′-3′)** |
| --- | --- |
| **Primers for clone of *FtUFGTs*** | |
| *FtUFGT6-F* | ATGAATCCCAATCGCCATGAA |
| *FtUFGT6-R* | CTTTTGACAATGCCGTGATCTTAC |
| *FtUFGT7-F* | ATGGCAACCCCTCTTCATGTAG |
| *FtUFGT7-R* | TTTCCTACCAGCAACTTTCATGTAC |
| *FtUFGT8-F* | ATGTCTGCCCCAATTCACGTA |
| *FtUFGT8-R* | TGGTGAATTTGTCCTAGGAGCA |
| *FtUFGT9-F* | ATGAACCCGAATCTCTCCATCA |
| *FtUFGT9-R* | CTTGTCACTCAACTTCTTCTTCACG |
| *FtUFGT15-F* | ATGGGATCGGAGAATCAAACAG |
| *FtUFGT15-R* | AGCCCTTAACTCCTTCACAAATTC |
| *FtUFGT40-F* | ATGCAGAAGCGGGTCTTCC |
| *FtUFGT40-R* | ATCAACAACTCCATGTTGAGAAAC |
| *FtUFGT41-F* | ATGACATCATCGACGGGAGG |
| *FtUFGT41-R* | CATGACCGCGGCCACC |
| **Primers for real-time quantitative PCR** | |
| *qFtUFGT6-F* | TGGAAGGTGTTTGTGGAGGTG |
| *qFtUFGT6-R* | CGTTGCCCATACTTTCCACTGT |
| *qFtUFGT7-F* | CGGGCATTGTCTTGTAGTGTTG |
| *qFtUFGT7-R* | CAGTGCTCGTGCTATGTCGTCT |
| *qFtUFGT8-F* | TCACAGATTTCGCCTCTTATTGG |
| *qFtUFGT8-R* | TCTTACCCTAACTCGTCCCTCGT |
| *qFtUFGT9-F* | AAGCCCGATTCTGACCCGT |
| *qFtUFGT9-R* | TTCCAGCATTGCCAACAGATT |
| *qFtUFGT15-F* | TTGGAGATGAAGTTGAGTCAGTTTG |
| *qFtUFGT15-R* | TTGCAGGGAATTGGTCAGGTAC |
| *qFtUFGT40-F* | TATCGCACAAGGCGGTAGGT |
| *qFtUFGT40-R* | GCACCAACACTCACACCAATACC |
| *qFtUFGT41-F* | AAGCTATAAGCGAAGGCGGAA |
| *qFtUFGT41-R* | CGAGTTGGAGACATCTTTGGAGT |
| *qFtH3-F* | GAAATTCGCAAGTACCAGAAGAG |
| *qFtH3-R* | CCAACAAGGTATGCCTCAGC |
| **Primers for constructing expression vector** | |
| *FtUFGT6-pCHF3-F* | GAGCTCGGTACCCGGGGATCCATGAATCCCAATCGCCATGAA |
| *FtUFGT6-pCHF3-R* | TCCAAGGGCGAATTGGTCGACCTTTTGACAATGCCGTGATCTTAC |
| *FtUFGT7-pCHF3-F* | GAGCTCGGTACCCGGGGATCCATGGCAACCCCTCTTCATGTAG |
| *FtUFGT7-pCHF3-R* | TCCAAGGGCGAATTGGTCGACTTTCCTACCAGCAACTTTCATGTAC |
| *FtUFGT8-pCHF3-F* | GAGCTCGGTACCCGGGGATCCATGTCTGCCCCAATTCACGTA |
| *FtUFGT8-pCHF3-R* | TCCAAGGGCGAATTGGTCGACTGGTGAATTTGTCCTAGGAGCA |
| *FtUFGT9-pCHF3-F* | GAGCTCGGTACCCGGGGATCCATGAACCCGAATCTCTCCATCA |
| *FtUFGT9-pCHF3-R* | TCCAAGGGCGAATTGGTCGACCTTGTCACTCAACTTCTTCTTCACG |
| *FtUFGT15-pCHF3-F* | GAGCTCGGTACCCGGGGATCCATGGGATCGGAGAATCAAACAG |
| *FtUFGT15-pCHF3-R* | TCCAAGGGCGAATTGGTCGACAGCCCTTAACTCCTTCACAAATTC |
| *FtUFGT40-pCHF3-F* | GAGCTCGGTACCCGGGGATCCATGTCCACCGCCAACGC |
| *FtUFGT40-pCHF3-R* | TCCAAGGGCGAATTGGTCGACATCAACAACTCCATGTTGAGAAAC |
| *FtUFGT41-pCHF3-F* | GAGCTCGGTACCCGGGGATCCATGACATCATCGACGGGAGG |
| *FtUFGT41-pCHF3R* | TCCAAGGGCGAATTGGTCGACCATGACCGCGGCCACC |
| **Primers for clone of *FtUFGTs* promoter** | |
| *pFtUFGT6-F* | TGTACCAAATCTTTTTTTAATCTTCAAT |
| *pFtUFGT6-R* | CTCTTTTCTGATGCGATATTGTGT |
| *pFtUFGT7-F* | GAATAAATGTTGATAATAGTCTTTTAAT |
| *pFtUFGT7-R* | TTTCGTGTGATGAGTACAATAATA |
| *pFtUFGT8-F* | AACAACAGTCTGTGGTTGCCTTAT |
| *pFtUFGT8-R* | CCCTATTTATACAAAACAATATTTCCTC |
| *pFtUFGT9-F* | TTAAACATTCGTAAGATGAAATAACCT |
| *pFtUFGT9-R* | AATCGGACGGCATAGATAGGAGGGAG |
| *pFtUFGT15-F* | TTCGCGCGACGTGGCTATAGTGA |
| *pFtUFGT15-R* | ACCACTACCACATGGGGGACA |
| *pFtUFGT40-F* | ATCGTAAACTTACTTGAATATGCGT |
| *pFtUFGT40-R* | GGGTACTTGGAGGATTTGGATA |
| *pFtUFGT41-F* | CTCGATAAACCCATTTTGATATTTTT |
| *pFtUFGT41-R* | AGCTGACGTAACCTTTTGTTTTTT |
|  |  |
